# Editorial: Organ Perfusion and Oxygenation in the Sick Infant

**DOI:** 10.3389/fped.2021.840917

**Published:** 2022-01-27

**Authors:** Elisabeth M. W. Kooi, Arend F. Bos, Jonathan P. Mintzer

**Affiliations:** ^1^Division of Neonatology, Beatrix Children's Hospital, University Medical Center Groningen, University of Groningen, Groningen, Netherlands; ^2^Mountainside Medical Center, Montclair, NJ, United States

**Keywords:** organ oxygenation, neonate, perfusion, NIRS (Near-infrared Spectroscopy), non-invasive cardiac output monitor (NICOM)

## Introduction

In neonates, a variety of postnatal diseases affect organ perfusion and oxygenation during the first weeks of life. During this critical period, preterm infants are highly susceptible to impaired organ blood flow and oxygen delivery, potentially resulting in end-organ injury. In addition, infants with intrauterine growth restriction, perinatal hypoxia, congenital heart disease, or requiring cardiothoracic interventions are similarly at risk for cerebral and/or peripheral organ hypoxic-ischemic injury. Such perturbations during the earliest stages of life are associated with significant neonatal mortality risks and an elevated likelihood of neurodevelopmental impairment among survivors (1).

Conventionally, indirect systemic measures, including heart rate, blood pressure (BP), and pulse oximetry, are monitored to guide the clinical management of sick neonates. In addition, biochemical measurements, including pH, pO_2_, and lactate, aid neonatologists in assessment of systemic hemodynamics in these infants. However, these indirect monitoring techniques provide information generated after organ injury has already begun, at which point reactive therapeutic maneuvers may be conducted (2).

The ability to assess real-time individual tissue oxygenation, perfusion, and oxygen extraction may help clinicians recognize infants at risk for hypoxic-ischemic organ injury. Indeed, such monitoring may result in earlier management of varied neonatal disease states, including hypoxic-ischemic encephalopathy, clinically significant anemia, and necrotizing enterocolitis, among others. Furthermore, tissue oxygenation monitoring could also provide information concerning regulation of tissue perfusion and/or tissue metabolic demand, thus providing enhanced insight into the pathogenesis of various neonatal disease states.

Over the last decades, there has been increasing interest in various non-invasive modalities to assess macro- and micro-perfusion among hemodynamically unstable newborn infants (3). Near-infrared spectroscopy (NIRS) seems a promising tool to non-invasively assess individual tissue oxygenation, but also has its limitations in clinical practice. In addition, non-invasive assessment of cardiac output also demonstrates promise as a method to improve understanding of organ blood flow among critically ill neonates.

We present a series of articles on this topic concerning the use of NIRS and non-invasive cardiac output measurements to assess organ perfusion and oxygenation in the sick infant.

## Cerebral Oxygen Saturation Measured by NIRS

Ongoing research into neonatal cerebral oxygenation, either as a factor preceding the development of intraventricular hemorrhage or neurodevelopmental impairment, or as an outcome measure of various clinical parameters, has resulted in several papers. The need for cerebral measurements is supported by Pazandak et al., who demonstrated in neonates with hypoxic-ischemic encephalopathy that BP-based requirements for dopamine and severity of brain injury were not associated. The authors suggest that BP measures alone provide an inadequate assessment of cerebral perfusion.

### Anemia and Red Blood Cell Transfusion

Kalteren et al. provided a systematic review of the literature which revealed that both anemia and red blood cell (RBC) transfusions are associated with cerebral oxygenation changes, brain injury, and neurodevelopmental impairment in preterm infants. The authors suggest to further investigate the role of cerebral oxygen saturation as a potential contributor to anemia-related neurodevelopmental outcomes in the individualized treatment of neonatal anemia.

Pritišanac et al. concluded from their literature review that, although from limited data, it seems that lowering the fraction of fetal Hb decreases peripheral muscle oxygen extraction, suggesting improvement in oxygen availability after administering adult packed red blood cells.

### Glucose, Lactate, and Inflammation

Besides hemoglobin, other factors may affect cerebral oxygenation in infants. Mattersberger et al. studied the literature on glucose and lactate levels in relation to cerebral oxygen saturation and confirmed a negative correlation between blood glucose level and cerebral oxygenation but found only one study with a negative correlation between blood lactate and cerebral oxygen saturation.

In a small cohort of preterm infants, Wolfsberger et al. demonstrated that fetal inflammation defined as elevated umbilical cord blood interleukin-6 values was associated with higher cerebral oxygen extraction shortly after birth. The simultaneous lower SpO_2_ and heart rate suggested potentially compromised cerebral oxygen delivery in cases of fetal inflammation.

### Cerebrovascular Autoregulation, IVH, Ductal Ligation

Cimatti et al. demonstrated that changes in cerebral oxygen saturation, possibly resulting from impaired cerebrovascular autoregulation during the transitional period, preceded the development of IVH in preterm infants. This finding supports previously published reports on the relation between impaired autoregulation measured using NIRS and cerebral hemorrhage in preterm infants (4, 5). Cerebral autoregulation also seems challenged during and after ductal ligation in a small cohort of preterm infants, especially when a posterolateral thoracotomy is performed as compared with sternotomy (Kooi et al.). The authors speculate that pulmonary venous return is reduced using the lateral approach, resulting in reduced cardiac output with subsequent cerebral perfusion pressures below the lower autoregulatory limit.

Michel-Macías et al. noticed from two cases and associated literature review that a drop in somatic NIRS values may precede clinical signs of hypoperfusion. They suggest that adding somatic NIRS monitoring to the assessment of cerebral oxygenation after ductal ligation may enhance early identification of post-ligation cardiac syndrome and guide interventions at earlier stages. Further prospective investigation is required to explore this further.

### Fetal Growth Restriction

Dix et al. demonstrated that fetal growth restricted (FGR) infants are less likely to demonstrate cerebral vasoconstrictive responses following noxious stimuli, possibly as a result from brain-sparing mechanisms. FGR infants appeared to have lower stroke volume and cerebral oxygenation during the second and third postnatal weeks. Richter et al. found that brain-sparing was associated with higher cerebral oxygenation after birth and enhanced behavior and executive function at 2 years of age. However, brain-sparing was negatively associated with performance IQ. Combining both observational studies by Dix and Richter, we speculate that cardiac function affected by FGR overrules postnatal brain-sparing with increasing postnatal age (6), and either early cerebral hyperoxia or later hypoxia may be associated with neurodevelopment.

## Somatic Oxygen Saturation Measured by NIRS

Assessment of somatic tissue oxygenation may aid in detecting local and/or systemic ischemia early. Some applications of renal and intestinal NIRS are discussed below.

### Renal Oxygenation

Harer and Chock discussed using NIRS for the assessment of renal oxygenation to predict or prevent acute kidney failure (AKI) in sick neonates. A review of the literature indeed suggests significant promise for renal oxygenation trending to prevent or decrease severity of AKI.

Alternatively, the assessment of both cerebral and renal oxygenation allows one to measure systemic hemodynamic effects of interventions. Terstappen et al. analyzed multi-site oxygenation data in a subgroup of infants born in the Dutch Strider Trial, which investigated the effect of prenatal sildenafil in FGR. The data suggested that prenatal sildenafil lowered renal but not cerebral oxygenation during the first 72 postnatal hours. The observed changes in renal oxygenation could reflect a vasoconstrictive rebound from sildenafil, as the authors hypothesized. Similar changes observed in accompanying vital parameters supported this hypothesis.

### Intestinal Oxygenation

NIRS could also aid in detecting attenuated intestinal oxygenation in preterm infants, as Dotinga et al. concluded from the literature. They found that the preterm intestine possesses a decreased “reserve” in cases of impaired intestinal oxygen delivery, as intestinal oxygen extraction may already be exhausted under stable conditions. A better understanding of the balance of intestinal oxygen supply and demand, perhaps assessed using NIRS, may help in guiding clinical management to prevent intestinal tissue hypoxia, as is suggested by the authors.

## Non-Invasive Cardiac Output Monitoring

Another tool to assess neonatal circulation is non-invasive cardiac output monitoring (NICOM). In the review by O'Neill et al., the authors evaluated the current utility of NICOM. They conclude that regardless of structural differences with echocardiographic derived cardiac output (CO), this approach is increasingly being used in various research settings. Its non-invasive nature informs the clinician continuously on CO trends, and could potentially impacts management and patient outcomes. However, before implementation in clinical practice, the effect of its use on clinical outcomes needs to be further addressed in future research.

## Conclusion

This series of cohort observations and literature reviews on cerebral and somatic oxygenation and perfusion assessment using NIRS and NICOM adds to the increasing evidence and knowledge base on the value of these tools to augment current neonatal hemodynamic monitoring practices. Moreover, this series contributes to our understanding of the role of hypoxia-ischemia in several neonatal diseases. A major advantage of NIRS and NICOM are their non-invasive nature allowing for the continuous assessment of trends in neonatal hemodynamics. Lack of gold standards for end-organ perfusion and oxygenation assessment, however, hampers validation of both tools and the absolute values these devices present need to be interpreted with caution. However, the clinical application of the more reliable trend-observation in end-organ perfusion and oxygenation, in addition to conventional hemodynamic monitoring, will hopefully result in improved care for the sick newborn. Further investigation is clearly warranted.

## Author Contributions

EK, AB, and JM edited the papers discussed in this editorial. EK wrote the first draft of the editorial. AB and JM edited this version into the current version. All authors approved submission of this paper.

## Conflict of Interest

The authors declare that the research was conducted in the absence of any commercial or financial relationships that could be construed as a potential conflict of interest.

## Publisher's Note

All claims expressed in this article are solely those of the authors and do not necessarily represent those of their affiliated organizations, or those of the publisher, the editors and the reviewers. Any product that may be evaluated in this article, or claim that may be made by its manufacturer, is not guaranteed or endorsed by the publisher.
